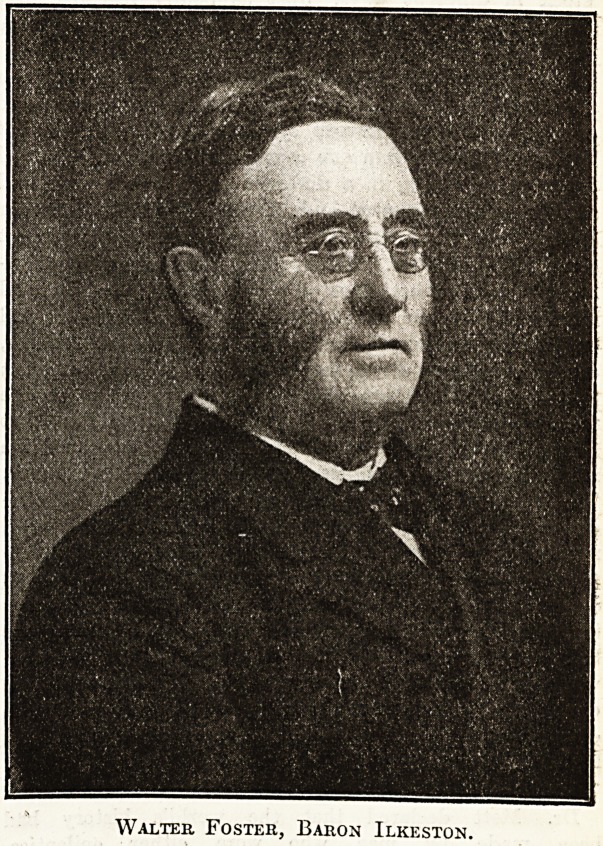# Medicine and the House of Lords: The Death of Lord Ilkeston

**Published:** 1913-02-08

**Authors:** 


### MEDICINE AND THE HOUSE OF LORDS.

### The Death of Lord Ilkeston.

A month ago we intimated in these columns that Lord Ilkeston's health was causing the very gravest anxiety to his family and friends; now it is our sorrowful duty to chronicle his decease. As a matter of fact, it has been known for a year and more that his active career was terminated, and that a malignant growth had proved to be beyond the reach of surgical succour.

Balthazar Walter Foster, first Baron Ilkeston, was born seventy-two years ago at Cambridge. Although both his parents were English, he was brought up in Ireland, where both his school and college education were conducted. After graduating at Trinity College, Dublin, Foster went to Birmingham at the age of twenty-one as medical tutor to Queen's College and assistant physician to the Queen's Hospital. At that time, it is scarcely necessary to say, Birmingham by no means enjoyed the reputation as a teaching centre that it has to-day; otherwise one so young could hardly have aspired to the positions he attained, and even at that time the achievement shows how highly his abilities were esteemed by his seniors. For a time he was Professor of Anatomy, and in 1869 became Professor of Medicine and physician in the Birmingham General Hospital, whose creation out of smaller and less important institutions he had largely helped to bring about.

Teaching, educational work, and a large private practice would have filled up the time and absorbed the energies of many men in his place. But Birmingham in the 'seventies knew Joseph Chamberlain in his early prime, and the strenuous life was in the atmosphere of the city. Foster served six years upon the Municipal Council, and contrived to find time also for medical journalism as a member of the *Lancet* staff. In 1885 Dr. Foster entered Parliament as a Liberal of advanced views and as member for the mining constituency of Ilkeston, Derbyshire. Among his constituents he inspired affection and confidence in a quite remarkable degree, and his contested elections resulted in enormous majorities in his favour. In the Liberal Ministry of 1892-95 he was Parliamentary Secretary to the Local Government Board. In this capacity his wide experience of medical and public health matters stood him in such good stead that it can only be marvelled at that anyone but a doctor should ever have been appointed to the post since.

For the ten years—1886-1896—Sir Walter Foster, as he had now become, was one of the direct representatives of the profession on the General Medical Council. In recognition of these and his many other services to the profession in Birmingham and London, and of his work as a parliamentarian and a minister, Sir Walter Foster was in 1897 the recipient of a gold medal from the British Medical Association. As we recalled in a recent issue (December 28, 1912) he wrote some thirty years ago upon "The political powerlessness of the medical profession," a powerlessness which he has just lived to see fully exemplified. It is always dangerous to speculate upon what might have happened had circumstances been different; but Lord Ilkeston's personal popularity with his party, his strong common sense and robust methods of expressing it, together with his knowledge of all sides of medical practice, might have helped to soften the heart of the Chancellor had his Lordship been in good health and full vigour during the troublous times of the last eighteen months.

When the Liberal Party returned to power in 1906, Sir Walter was one of the few remaining who had held office in the last Liberal Government.

Walter Foster, Baron Ilkeston.

For some reason he was passed over by Sir Henry Campbell-Bannerman, an omission which many good judges regarded as extremely foolish in the interests both of the party and of the country. He was, however, made a Privy Councillor, and four years later he accepted a peerage at a time when a safe seat was badly needed for Colonel Seely. To the last Lord Ilkeston remained one of the advanced wing of his party; but it is more as an accomplished physician, a genial companion, a man of robust common sense and of indefatigable industry that his memory will live long in the land and in the institutional world. To his many friends in both political parties his death will be felt as a personal loss. He will long remain a shining example of the value of medical men in public life, so long, at any rate, as the statesman is preferred to the party politician.

## Figures and Tables

**Figure f1:**